# Antigenic characterization of SARS-CoV-2 Omicron subvariants XBB.1.5, BQ.1, BQ.1.1, BF.7 and BA.2.75.2

**DOI:** 10.1038/s41392-023-01391-x

**Published:** 2023-03-15

**Authors:** Airu Zhu, Peilan Wei, Miao Man, Xuesong Liu, Tianxing Ji, Jiantao Chen, Canjie Chen, Jiandong Huo, Yanqun Wang, Jincun Zhao

**Affiliations:** 1 State Key Laboratory of Respiratory Disease, National Clinical Research Center for Respiratory Disease, Guangzhou Institute of Respiratory Health, the First Affiliated Hospital of Guangzhou Medical University, Guangzhou, China; 2 University Hospital and Center for Biomedicine and Innovations, Faculty of Medicine, Macau University of Science and Technology, Macau SAR, China; 3 Clinical Laboratory Medicine Department, The Second Affiliated Hospital of Guangzhou Medical University, Guangzhou, China; 4 Guangzhou laboratory, Bio-island, Guangzhou, China; 5 Shanghai Institute for Advanced Immunochemical Studies, School of Life Science and Technology, ShanghaiTech University, Shanghai, China

**Keywords:** Infection, Vaccines

**Dear Editor,**

Recently, a number of new Omicron subvariants related to BA.4/5 and BA.2.75 have emerged and shown remarkable antibody evasion capacities, in particular BF.7, BQ.1, BQ.1.1, BA.2.75.2, XBB and XBB.1.5.^1^ Unsurprisingly, these new subvariants are quickly gaining prevalence worldwide. In fact, some of them have outcompeted BA.5 in the USA according to CDC’s national genomic surveillance data in which, as of 6^th^ February 2023, XBB.1.5, BQ.1.1, BQ.1, XBB and BF.7 have achieved a dominance of 66.4%, 19.9%, 7.3%, 2.3% and 0.5% in the USA, as compared to 0.5% for BA.5. In this report, using plasma samples collected from individuals following different vaccination strategies and COVID-19 convalescent donors, we performed pseudoviral neutralization assays to confirm severe reductions in neutralization titers against BF.7, BQ.1, BQ.1.1, BA.2.75.2, XBB and XBB.1.5 in comparison to other Omicron sub-lineages. XBB and XBB.1.5 were shown to be remarkably resistant to plasma neutralization in all tested cohorts. By comparing the differential neutralization profiles, we found that a heterologous booster with an aerosolized vaccine following 2 doses of inactivated vaccine seemed to be superior to other vaccination strategies.

To evaluate the antibody evasion capacity of the new variants, we constructed a panel of pseudotyped vesicular stomatitis virus (VSV)^2^ expressing the S gene from BF.7, BQ.1, BQ.1.1, BA.2.75.2, XBB and XBB.1.5 and other SARS-CoV-2 variants together with early pandemic wild type (WT) strain, used as a control. We first accessed the neutralization profile for plasma samples collected 4–6 weeks following symptom onset from unvaccinated convalescents infected with WT (WC group, *n* = 15) or Delta (DC group, *n* = 17), or plasma collected from vaccinees who had received 2 doses of inactivated vaccine CoronaVac (BA.2 group, *n* = 17) following BA.2 breakthrough infection or those who had received 3 doses of inactivated vaccine CoronaVac (BA.5 group, *n* = 19) following BA.5 breakthrough infection (Fig. 1a). Neutralization titers against BF.7, BQ.1, BQ.1.1, BA.2.75.2, XBB and XBB.1.5 were below or close to the limit of detection [given an arbitrary pVNT_50_ (the reciprocal dilution of plasma that neutralizes 50% of the input virus) value of 30] in both the WC and DC groups, although the titers to BA.2 and BA.4/5 were comparably low in both groups (Fig. 1b). In the BA.2 and BA.5 group, XBB and XBB.1.5 remained resistant to neutralization by plasma, but the titers against other variants were markedly increased as compared to the WC and DC group (Fig. 1b). Titers against BF.7, BQ.1, BQ.1.1, BA.2.75.2, XBB and XBB.1.5 were 3.2 to 9.8-fold lower than BA.4/5 in the BA.2 group, and 3.7 to 14.5-fold lower than BA.4/5 in the BA.5 group respectively.

**Fig. 1:**
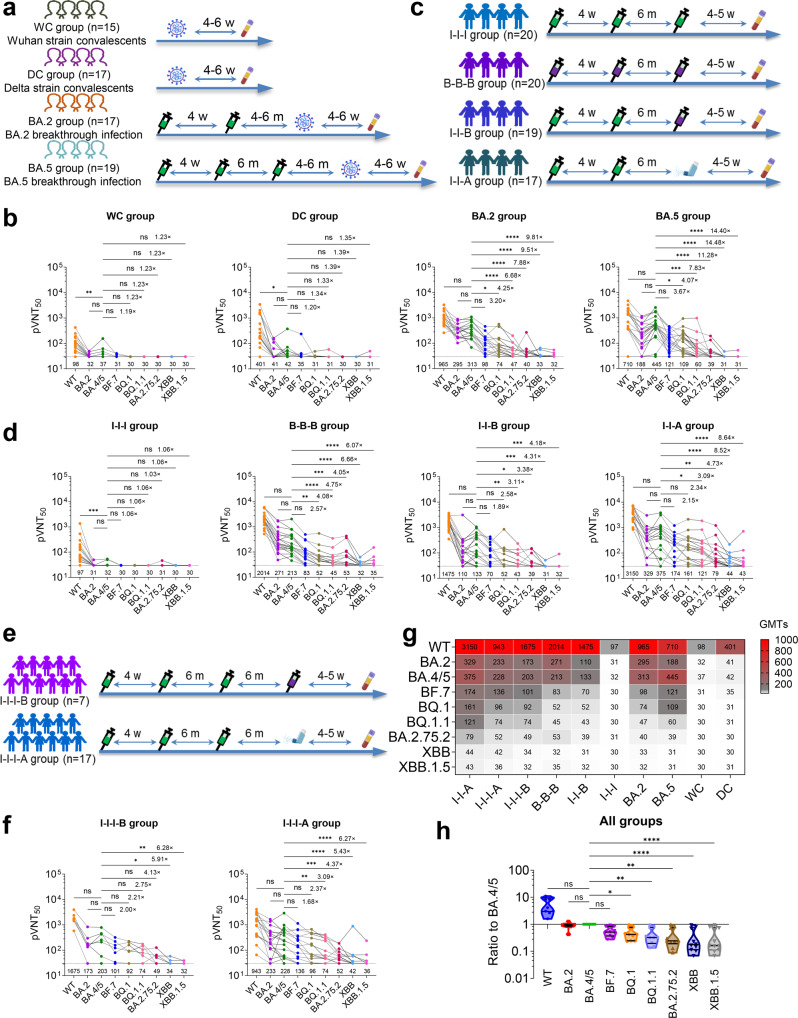
Plasma neutralization titers against Omicron variants in convalescents, BA.2 and BA.5 breakthrough infection and vaccinees. **a** Grouping information and timing of plasma sample acquisition from convalescents, BA.2 and BA.5 breakthrough infection patients, w represented week, m represented month. **b** Neutralizing titers against various SARS-CoV-2 pseudovirus in plasma from convalescents from prototype or Delta SARS-CoV-2 (WC, DC) and Omicron BA.2 or BA.5 breakthrough infection groups (BA.2, BA.5). **c** Grouping information and timing of plasma sample acquisition from vaccinees who received homologous (I-I-I, B-B-B) or heterologous (I-I-B, I-I-A) booster vaccination. I represented an inactivated vaccine CoronaVac, B represented an mRNA vaccine BNT162b2, and A represented an aerosolized vaccine Ad5-nCoV. **d** Neutralizing titers against various SARS-CoV-2 pseudovirus in plasma from vaccinees in homologous or heterologous COVID-19 booster vaccination groups as described in panel **c**. **e** Grouping information and timing of plasma sample acquisition from vaccinees who received a second booster vaccination (I-I-I-B, I-I-I-A). **f** Neutralizing titers against various SARS-CoV-2 pseudovirus in plasma from vaccinees receiving second COVID-19 booster vaccination as described in panel **e**. In panel **b**, **d** and **f**, SARS-CoV-2 pseudovirus used for neutralizing assay included WT, BA.2, BA.4/5, BF.7, BQ.1, BQ.1.1, BA.2.75.2, XBB and XBB.1.5. The geometric mean neutralizing titers (GMTs) were shown at the bottom in each panel, and fold changes of GMTs against Omicron BF.7, BQ.1, BQ.1.1, BA.2.75.2, XBB and XBB.1.5 relative to BA.4/5 were labeled. **g** Comparison of immune escape properties against diverse Omicron subvariants from vaccinees, convalescents and breakthrough infection were summarized in the heatmap of GMTs. **h**. The immune escape assessments of different variants were performed as the ratio of their GMTs to that of BA.4/5. Data distribution was confirmed with Shapiro-Wilk normality test, Friedman test with Dunn’s multiple comparisons test and Kruskal–Wallis test with Dunn’s multiple comparisons test were used for evaluating differences among the experimental groups. *p* values are displayed as ns for *p* > 0.05, *p < 0.05, **p < 0.01, ***p < 0.001, and ****p < 0.0001

Vaccine plasma were taken from four different groups of individuals, including the I-I-I group (vaccinees who had received 3 doses of inactivated vaccine CoronaVac, *n* = 20), the B-B-B group (vaccinees who had received 3 doses of mRNA vaccine BNT162b2, *n* = 20), the I-I-B group (vaccinees who had received 2 doses of inactivated vaccine CoronaVac followed by a heterologous booster with mRNA vaccine BNT162b2, *n* = 19) and the I-I-A group (vaccinees who had received 2 doses of inactivated vaccine CoronaVac followed by a heterologous booster with aerosolized vaccine Ad5-nCoV, *n* = 17) (Fig. 1c). The I-I-I group showed a very similar profile to that observed in the WC group (Fig. 1d) such that only low neutralization titers [geometric mean pVNT_50_ = 97] were elicited against WT and responses against the Omicron subvariants were below or close to the limit of detection. By contrast, much higher titers were induced in the B-B-B group (Fig. 1d). While the tripled dosed inactivated virus vaccination performed poorly, sequential vaccination of two doses of inactivated vaccine and a single dose of mRNA vaccine or aerosolized vaccine substantially increased the neutralization titers against the new subvariants (Fig. 1d). As observed in the BA.2 and BA.5 group, neutralization titers against the new variants were consistently higher for BF.7, followed by BQ.1, BQ.1.1, BA.2.75.2, XBB and XBB.1.5 in B-B-B, I-I-B and I-I-A group.

Next, neutralization assays were performed using plasma samples obtained from vaccinees who had received 3 doses of inactivated vaccine CoronaVac followed by a heterologous booster with mRNA vaccine BNT162b2 (I-I-I-B group, *n* = 7) or aerosolized vaccine Ad5-nCoV (I-I-I-A group, *n* = 17) (Fig. 1e). The neutralization profile for the two groups are similar (Fig. 1f), the new subvariants showed greater resistance than BA.4/5 in both groups, with a 2.0 to 6.3-fold reduction in titers in the I-I-I-B group and a 1.7 to 6.3-fold reduction in the I-I-I-A group, except that the I-I-I-A strategy elicited lower titers against the WT strain compared to I-I-I-B. In fact, not only for the WT strain, the neutralization titers induced by BF.7, BQ.1, BQ.1.1, BA.2.75.2, XBB and XBB.1.5 were consistently lower in the I-I-I-A group compared to I-I-A where a booster with aerosolized vaccine was administered following two doses of inactivated vaccine rather than three doses (Fig. 1g). Considering the comparable age and sex distribution between these two groups, the difference may be caused by the vaccination strategies. According to a new study,^3^ pre-existing high-affinity antibodies would inhibit immune responses by lowering the activation threshold for B cells and direct masking of their cognate epitopes, thus B cell responses induced by the heterologous Ad5-nCoV booster vaccine may be dampened by a higher pre-existing high-affinity antibody levels in I-I-I-A individuals when compared to the I-I-A ones. Similar trends were observed for both vaccine- and infection-induced plasma, regardless of the vaccination status (Fig. 1 g, h), enhanced neutralization resistance of SARS-CoV-2 Omicron subvariants BF.7, BQ.1, BQ.1.1, BA.2.75.2, XBB and XBB.1.5 was observed when compared with their parent BA.2 and BA.4/5. Multiple vaccination strategies, including I-I-I, B-B-B, I-I-B, I-I-I-B, I-I-A and I-I-I-A, failed to elicit high neutralizing antibody titer against the newly emerged Omicron subvariant and the rank of neutralization evasion is in the order of BA.2/BA.5 < BF.7 < BQ.1 < BQ.1.1 < BA.2.75.2 < XBB/XBB.1.5, especially XBB/XBB.1.5 which shows superior antibody escaping capability. Consistent to our results, antibody evasion to new subvariants BA.2.75.2, BQ.1.1, XBB.1.5, CH.1.1, and CA.3.1 have been reported in parental mRNA vaccine or BA.5-bivalent booster,^4,5^ calling urgently for new bivalent vaccines and better-off vaccination strategies.

In summary, we study the neutralization of these new subvariants using a range of plasma samples from natural and breakthrough infections, as well as homologous and heterologous vaccinations. Compared to BA.5, the new subvariants showed stronger antibody escape in all tested cohorts, and the rank of neutralization evasion is in the order of BA.2/BA.4/5 < BF.7 < BQ.1 < BQ.1.1 < BA.2.75.2 < XBB/XBB.1.5 based on the geometric mean neutralizing titers (GMTs). Notably, neutralization activity was exceptionally low against XBB/XBB.1.5 in all cases. Whilst triple-dosed inactivated vaccine elicited very low neutralizing antibody responses against the Omicron subvariants, a heterologous booster with an aerosolized vaccine or an mRNA vaccine following 2 or 3 doses of inactivated vaccine substantially improved the neutralization profiles, although taking a heterologous booster of aerosolized vaccine following 2 doses of inactivated vaccine seemed to generate superior results to others. Our study thus provides valuable information that may help to guide the design of vaccination strategy.

## Supplementary information

supplemental material

## Data Availability

The data and materials used in the current study are available from the corresponding authors upon reasonable request.

## References

[1] Tuekprakhon A (2022). Antibody escape of SARS-CoV-2 Omicron BA.4 and BA.5 from vaccine and BA.1 serum. Cell..

[2] Nie J (2020). Quantification of SARS-CoV-2 neutralizing antibody by a pseudotyped virus-based assay. Nat. Protoc..

[3] Schaefer-Babajew D (2023). Antibody feedback regulates immune memory after SARS-CoV-2 mRNA vaccination. Nature..

[4] Kurhade C (2023). Low neutralization of SARS-CoV-2 Omicron BA.2.75.2, BQ.1.1 and XBB.1 by parental mRNA vaccine or a BA.5 bivalent booster. Nat. Med..

[5] Qu, P. et al. Extraordinary Evasion of Neutralizing Antibody Response by Omicron XBB.1.5, CH.1.1 and CA.3.1 Variants. Preprint at 10.1101/2023.01.16.524244 (2023).

