# Supplementary material for: Antigenic characterization of SARS-CoV-2 Omicron subvariants XBB.1.5, BQ.1, BQ.1.1, BF.7 and BA.2.75.2
Source: Signal Transduct Target Ther. 2023 Mar 15;8:125. doi: 10.1038/s41392-023-01391-x (PMC10015517; doi:10.1038/s41392-023-01391-x)
Supplement: Supplementary file 1 — supplemental material [file 41392_2023_1391_MOESM1_ESM.docx]

Supplementary Materials for

**Antigenic characterization of SARS-CoV-2 Omicron subvariants XBB.1.5, BQ.1, BQ.1.1, BF.7 and BA.2.75.2**

Airu Zhu^1#^, Peilan Wei^1#^, Miao Man^2#^, Xuesong Liu^1#^, Tianxing Ji^3#^, Jiantao Chen^1^, Canjie Chen^1^, Jiandong Huo^1*^, Yanqun Wang^1*^, Jincun Zhao^1,4,5*^

^1^State Key Laboratory of Respiratory Disease, National Clinical Research Center for Respiratory Disease, Guangzhou Institute of Respiratory Health, the First Affiliated Hospital of Guangzhou Medical University, Guangzhou, China; ^2^University Hospital and Center for Biomedicine and Innovations, Faculty of Medicine, Macau University of Science and Technology, Macau SAR, China; ^3^Clinical Laboratory Medicine Department, The Second Affiliated Hospital of Guangzhou Medical University, Guangzhou, China; ^4^Guangzhou laboratory, Bio-island, Guangzhou, China; ^5^Shanghai Institute for Advanced Immunochemical Studies, School of Life Science and Technology, ShanghaiTech University, Shanghai, China

Correspondence to: Jincun Zhao (zhaojincun@gird.cn), Yanqun Wang (wangyanqun@gird.cn), Jiandong Huo (huojiandong@gird.cn)

**This PDF file includes:**

Materials and Methods

Tables S1 to S5

**Material and Methods**

**SARS-CoV-2 convalescents**

There were 15 and 17 plasma samples collected from COVID-19 convalescents recovered from prototype and delta SARS-CoV-2 infection, respectively (Supplementary Table. S1 and S2). These convalescent samples were collected 4-6 weeks after illness onset. Consent forms were signed prior to blood collection. Ethical approval was obtained from the Research Ethics Committee of the First Affiliated Hospital of Guangzhou Medical University.

**BA.2 and BA.5 breakthrough infection patients**

There were 17 plasma samples collected from Omicron BA.2 breakthrough infection patients, included 1 male and 16 females with ages ranging from 11 to 60 and all the BA.2 breakthrough patients have received 2-dose inactivated vaccine. Meanwhile, 19 plasma samples were collected from Omicron BA.5 breakthrough infection patients, included 7 males and 12 females with ages ranging from 28 to 70 (Supplementary Table. 1 and 2), all the BA.5 breakthrough infection patients have received 3-dose inactivated vaccine. All the plasma was collected at 4-6 weeks after breakthrough infection. Ethical approval was obtained from the Research Ethics Committee of the First Affiliated Hospital of Guangzhou Medical University.

**Homologous and heterologous COVID-19 booster vaccinations**

Multiple vaccine strategies were performed in this study, including homologous and heterologous booster vaccinations. In detail (Supplementary Table. S1 and S3), (1) inactivated vaccine homologous booster group, 20 donors received 3-dose inactivated vaccine (I-I-I group), included 6 males and 14 females with ages ranging from 23 to 58; (2) mRNA vaccine homologous booster group, 20 donors received 3-dose mRNA vaccine BNT162b2 (B-B-B group), included 8 male and 12 female with ages ranging from 21 to 43; (3) inactivated vaccine/mRNA vaccine heterologous booster group, 19 donors received 2-dose inactivated vaccine plus one dose mRNA vaccine (I-I-B group), included 6 male and 13 female with ages ranging from 20 to 63 and (4) 17 donors received 2-dose inactivated vaccine plus one dose aerosolized Ad5-nCoV (I-I-A group), included 6 males and 11 females with ages ranging from 22 to 49. All the plasma samples were collected at 4-5 weeks after the booster vaccination.

**The second COVID-19 booster shot**

Seven donors received 3-dose inactivated vaccine plus one dose mRNA vaccine (I-I-I-B group), included one male and 6 females with ages ranging from 37 to 72, and 17 donors received 3-dose inactivated vaccine plus one dose aerosolized Ad5-nCoV vaccine (I-I-I-A group), included 5 males and 12 females with ages ranging from 22 to 37 (Supplementary Table. S1 and S4). All the plasma samples were collected at 4-5 weeks after the second booster vaccination. This study was performed in strict accordance with human subject protection guidance proved by the Research Ethics Committee of the First Affiliated Hospital of Guangzhou Medical University.

**Production and titration of SARS-CoV-2 pseudovirus**

SARS-CoV-2 subvariants pseudotyped viruses were generated with vesicular stomatitis virus (VSV) pseudotyping system as previously described ^1,2^. In detail, plasmid encoding propotype or variants SARS-CoV-2 spike protein, including wild type (WT), BA.2, BA.4/5, BF.7, BQ.1, BQ.1.1, BA.2.75.2, XBB and XBB.1.5 (Supplementary Table. S5), were transfected into 293T cells, respectively, 24 h later transduced 293T cells were infected with rVSV-ΔG-luc, viral supernatant was harvested 24 h after infection and stored at −80°C (Guangzhou Darui Technology Co.,LTD). The 50% tissue culture infectious dose (TCID_50_) was calculated using the Reed–Muench method, as described previously^2^. In detail, to determine the titration of the SARS-CoV-2 pseudovirus, a 10-fold initial dilution was made in 96-well culture plates followed by serial 5-fold dilutions. Then, the white solid 96-well plates were seeded with trypsin-treated 293T-hACE2 cells. After 24 h incubation, 50 μL of luciferase substrate (Vazyme) was added to each well for the detection of luminescence using a microplate luminometer (Bioteck). The positive well was determined as ten-fold relative luminescence unit (RLU) values higher than the cell background.

**Neutralization assay based on a VSV pseudotyped system**

Neutralization was measured by the reduction in luc gene expression, as described previously^3^ for the VSV pseudovirus neutralization assay. 50 μL SARS-CoV-2 pseudovirus (3.0×10^4^ TCID_50_/mL) were incubated with 50 μL 3-fold serially diluted plasma at 37 °C for 1 hour, and then 100 μL cell suspensions of human 293T-hACE2 cells (5×10^5^ cells/mL) were added to the mixtures, experiments were performed in duplicate. After 24 hours’ incubation at 37°C, neutralizations potencies of plasma were evaluated in a luciferase assay. The 50% pseudovirus neutralization titer (pVNT_50_) was deﬁned as the plasma dilution at which the relative light units (RLUs) were reduced by 50% compared with the virus control wells (virus + cells) after subtraction of the background RLUs in the control groups with cells only.

**Statistical analysis**

Antibody responses were reported as the 50% pseudovirus neutralization titer (pVNT_50_), the pVNT_50_ value of pseudovirus neutralization activity below the limit of detection (1:30) was set to 1:30, pVNT_50_ value above the limit of detection (1:7290) was set to 1:7290. Statistical analyses were performed by using GraphPad Prism 8 software. Data distribution was confirmed with Shapiro-Wilk normality test, Friedman test with Dunn's multiple comparisons test and Kruskal–Wallis test with Dunn’s multiple comparisons test were used for evaluating differences among the experimental groups, statistical analysis of age and gender were performed by one-way ANOVA and chi-squared test, respectively. Significance thresholds: ns p > 0.05, * p < 0.05, ** p < 0.01, *** p < 0.001, and **** p < 0.0001.

**References**

1 Whitt, M. A. Generation of VSV pseudotypes using recombinant ΔG-VSV for studies on virus entry, identification of entry inhibitors, and immune responses to vaccines. *J Virol Methods*. **169**, 365-374, (2010).

2 Nie, J. *et al.* Quantification of SARS-CoV-2 neutralizing antibody by a pseudotyped virus-based assay. *Nat Protoc*. **15**, 3699-3715, (2020).

3 Nie, J. *et al.* Establishment and validation of a pseudovirus neutralization assay for SARS-CoV-2. *Emerg Microbes Infect*. **9**, 680-686, (2020).

**Table S1. Cohort demographics.**

**Table S2. Detailed demographic information of convalescents, BA.2 and BA.5 breakthrough infection groups in this study.**

| **Cohort** | **Participant** | **Gender** | **Age** | **COVID-19 symptoms** |
| --- | --- | --- | --- | --- |
| **WC group** | WC-1 | Male | 64 | critically severe |
|  | WC-2 | Male | 49 | critically severe |
|  | WC-3 | Male | 53 | critically severe |
|  | WC-4 | Male | 61 | critically severe |
|  | WC-5 | Male | 42 | critically severe |
|  | WC-6 | Male | 58 | critically severe |
|  | WC-7 | Male | 26 | critically severe |
|  | WC-8 | Male | 79 | critically severe |
|  | WC-9 | Female | 72 | critically severe |
|  | WC-10 | Female | 55 | critically severe |
|  | WC-11 | Male | 66 | critically severe |
|  | WC-12 | Male | 68 | critically severe |
|  | WC-13 | Female | 65 | critically severe |
|  | WC-14 | Female | 38 | critically severe |
|  | WC-15 | Female | 73 | critically severe |
| **DC group** | DC-1 | Male | 66 | critically severe |
|  | DC-2 | Male | 75 | critically severe |
|  | DC-3 | Male | 85 | critically severe |
|  | DC-4 | Female | 92 | critically severe |
|  | DC-5 | Male | 66 | critically severe |
|  | DC-6 | Male | 80 | critically severe |
|  | DC-7 | Male | 66 | critically severe |
|  | DC-8 | Male | 63 | critically severe |
|  | DC-9 | Female | 85 | critically severe |
|  | DC-10 | Male | 69 | critically severe |
|  | DC-11 | Male | 85 | critically severe |
|  | DC-12 | Male | 73 | critically severe |
|  | DC-13 | Female | 63 | critically severe |
|  | DC-14 | Male | 47 | critically severe |
|  | DC-15 | Female | 63 | critically severe |
|  | DC-16 | Male | 53 | critically severe |
|  | DC-17 | Male | 40 | mild |
| **BA.2 group** | BA2-1 | Female | 15 | mild |
|  | BA2-2 | Female | 15 | mild |
|  | BA2-3 | Female | 16 | mild |
|  | BA2-4 | Female | 16 | mild |
|  | BA2-5 | Female | 16 | mild |
|  | BA2-6 | Female | 15 | mild |
|  | BA2-7 | Female | 16 | mild |
|  | BA2-8 | Female | 16 | mild |
|  | BA2-9 | Female | 16 | mild |
|  | BA2-10 | Female | 16 | mild |
|  | BA2-11 | Female | 15 | mild |
|  | BA2-12 | Female | 16 | mild |
|  | BA2-13 | Female | 16 | mild |
|  | BA2-14 | Female | 17 | mild |
|  | BA2-15 | Female | 16 | mild |
|  | BA2-16 | Female | 60 | mild |
|  | BA2-17 | Female | 36 | mild |
| **BA.5 group** | BA5-1 | Female | 53 | mild |
|  | BA5-2 | Female | 50 | mild |
|  | BA5-3 | Male | 52 | mild |
|  | BA5-4 | Female | 55 | mild |
|  | BA5-5 | Female | 52 | mild |
|  | BA5-6 | Male | 32 | mild |
|  | BA5-7 | Male | 52 | mild |
|  | BA5-8 | Female | 40 | mild |
|  | BA5-9 | Female | 47 | mild |
|  | BA5-10 | Male | 56 | mild |
|  | BA5-11 | Female | 52 | mild |
|  | BA5-12 | Female | 69 | mild |
|  | BA5-13 | Female | 44 | mild |
|  | BA5-14 | Female | 37 | mild |
|  | BA5-15 | Female | 53 | mild |
|  | BA5-16 | Female | 28 | mild |
|  | BA5-17 | Female | 58 | mild |
|  | BA5-18 | Male | 70 | mild |
|  | BA5-19 | Male | 35 | mild |

**Table S3. Detailed demographic information of homologous or heterologous COVID-19 booster vaccinations groups in this study.**

| **Cohort** | **Participant** | **Gender** | **Age** |
| --- | --- | --- | --- |
| **I-I-I group** | I-I-I-1 | Female | 36 |
|  | I-I-I-2 | Female | 31 |
|  | I-I-I-3 | Female | 31 |
|  | I-I-I-4 | Female | 24 |
|  | I-I-I-5 | Female | 26 |
|  | I-I-I-6 | Female | 42 |
|  | I-I-I-7 | Female | 40 |
|  | I-I-I-8 | Male | 31 |
|  | I-I-I-9 | Male | 40 |
|  | I-I-I-10 | Female | 53 |
|  | I-I-I-11 | Female | 23 |
|  | I-I-I-12 | Female | 27 |
|  | I-I-I-13 | Male | 39 |
|  | I-I-I-14 | Female | 49 |
|  | I-I-I-15 | Female | 58 |
|  | I-I-I-16 | Male | 34 |
|  | I-I-I-17 | Female | 51 |
|  | I-I-I-18 | Female | 31 |
|  | I-I-I-19 | Male | 25 |
|  | I-I-I-20 | Male | 26 |
| **B-B-B group** | B-B-B-1 | Male | 42 |
|  | B-B-B-2 | Female | 22 |
|  | B-B-B-3 | Male | 28 |
|  | B-B-B-4 | Male | 27 |
|  | B-B-B-5 | Female | 34 |
|  | B-B-B-6 | Male | 43 |
|  | B-B-B-7 | Female | 31 |
|  | B-B-B-8 | Male | 34 |
|  | B-B-B-9 | Female | 32 |
|  | B-B-B-10 | Female | 32 |
|  | B-B-B-11 | Female | 26 |
|  | B-B-B-12 | Female | 21 |
|  | B-B-B-13 | Female | 34 |
|  | B-B-B-14 | Male | 39 |
|  | B-B-B-15 | Male | 43 |
|  | B-B-B-16 | Female | 28 |
|  | B-B-B-17 | Male | 27 |
|  | B-B-B-18 | Female | 39 |
|  | B-B-B-19 | Female | 35 |
|  | B-B-B-20 | Female | 33 |
| **I-I-B group** | I-I-B-1 | Female | 30 |
|  | I-I-B-2 | Female | 36 |
|  | I-I-B-3 | Female | 58 |
|  | I-I-B-4 | Male | 55 |
|  | I-I-B-5 | Female | 58 |
|  | I-I-B-6 | Female | 38 |
|  | I-I-B-7 | Female | 20 |
|  | I-I-B-8 | Female | 21 |
|  | I-I-B-9 | Female | 39 |
|  | I-I-B-10 | Female | 45 |
|  | I-I-B-11 | Female | 29 |
|  | I-I-B-12 | Female | 60 |
|  | I-I-B-13 | Female | 58 |
|  | I-I-B-14 | Male | 33 |
|  | I-I-B-15 | Female | 25 |
|  | I-I-B-16 | Male | 34 |
|  | I-I-B-17 | Male | 45 |
|  | I-I-B-18 | Male | 63 |
|  | I-I-B-19 | Male | 42 |
| **I-I-A group** | I-I-A-1 | Female | 23 |
|  | I-I-A-2 | Male | 23 |
|  | I-I-A-3 | Female | 25 |
|  | I-I-A-4 | Male | 22 |
|  | I-I-A-5 | Female | 23 |
|  | I-I-A-6 | Male | 43 |
|  | I-I-A-7 | Male | 43 |
|  | I-I-A-8 | Female | 46 |
|  | I-I-A-9 | Female | 35 |
|  | I-I-A-10 | Male | 22 |
|  | I-I-A-11 | Female | 26 |
|  | I-I-A-12 | Female | 27 |
|  | I-I-A-13 | Female | 44 |
|  | I-I-A-14 | Female | 26 |
|  | I-I-A-15 | Male | 49 |
|  | I-I-A-16 | Female | 27 |
|  | I-I-A-17 | Female | 34 |

**Table S4. Detailed demographic information of participants received 3-dose inactivated vaccine plus one dose mRNA vaccine or aerosolized Ad5-nCoV vaccine in this study.**

| **Cohort** | **Participant** | **Gender** | **Age** |
| --- | --- | --- | --- |
| **I-I-I-B group** | I-I-I-B-1 | Female | 44 |
|  | I-I-I-B-2 | Female | 43 |
|  | I-I-I-B-3 | Female | 64 |
|  | I-I-I-B-4 | Female | 50 |
|  | I-I-I-B-5 | Female | 37 |
|  | I-I-I-B-6 | Male | 72 |
|  | I-I-I-B-7 | Female | 71 |
| **I-I-I-A group** | I-I-I-A-1 | Female | 30 |
|  | I-I-I-A-2 | Female | 25 |
|  | I-I-I-A-3 | Female | 23 |
|  | I-I-I-A-4 | Female | 22 |
|  | I-I-I-A-5 | Female | 23 |
|  | I-I-I-A-6 | Male | 23 |
|  | I-I-I-A-7 | Male | 35 |
|  | I-I-I-A-8 | Female | 28 |
|  | I-I-I-A-9 | Female | 28 |
|  | I-I-I-A-10 | Female | 37 |
|  | I-I-I-A-11 | Male | 27 |
|  | I-I-I-A-12 | Male | 27 |
|  | I-I-I-A-13 | Female | 32 |
|  | I-I-I-A-14 | Female | 22 |
|  | I-I-I-A-15 | Female | 24 |
|  | I-I-I-A-16 | Female | 22 |
|  | I-I-I-A-17 | Male | 24 |

**Table S5. Reference accession number of SARS-CoV-2 wild type strain and mutations of Omicron subvariants BA.2, BA.5, BF.7, BQ.1, BQ.1.1, BA.2.75.2, XBB and XBB.1.5.**

| Pango lineages | Mutations in spike compared to WT |
| --- | --- |
| WT (NCBI accession number NC_045512.2) | NA |
| BA.2 | T19I, L24S, P25del, P26del, A27del，G142D，V213G，G339D，S371F，S373P，S375F，T376A，D405N，R408S，K417N，N440K，S477N，T478K，E484A，Q493R，Q498R，N501Y，Y505H，D614G，H655Y，N679K，P681H，N764K，D796Y，Q954H，N969K |
| BA.4/5 | T19I，L24S, P25del, P26del, A27del，H69Del, V70Del，G142D，V213G，G339D，S371F，S373P，S375F，T376A，D405N，R408S，K417N，N440K，L452R，S477N，T478K，E484A，F486V，Q498R，N501Y，Y505H，D614G，H655Y，N679K，P681H，N764K，D796Y，Q954H，N969K |
| BF.7 | T19I, L24S, P25del, P26del, A27del, H69Del, V70Del, G142D, V213G, G339D, R346T, S371F, S373P, S375F, T376A, D405N, R408S, K417N, N440K, L452R, S477N, T478K, E484A, F486V, Q498R, N501Y, Y505H, D614G, H655Y, N679K, P681H, N764K, D796Y, Q954H, N969K |
| BQ.1 | T19I, L24S, P25del, P26del, A27del, H69Del, V70Del, G142D, V213G, G339D, S371F, S373P, S375F, T376A, D405N, R408S, K417N, N440K, K444T, L452R, N460K, S477N, T478K, E484A, F486V, Q498R, N501Y, Y505H, D614G, H655Y, N658S, N679K, P681H, N764K, D796Y, Q954H, N969K |
| BQ.1.1 | T19I, L24S, P25del, P26del, A27del, H69Del, V70Del, G142D, V213G, G339D, R346T, S371F, S373P, S375F, T376A, D405N, R408S, K417N, N440K, K444T, L452R, N460K, S477N, T478K, E484A, F486V, Q498R, N501Y, Y505H, D614G, H655Y, N658S, N679K, P681H, N764K, D796Y, Q954H, N969K |
| BA.2.75.2 | T19I, L24S, P25del, P26del, A27del, G142D, K147E, W152R, F157L, I210V, V213G, G257S, G339H, R346T, S371F, S373P, S375F, T376A, D405N, R408S, K417N, N440K, G446S, N460K, S477N, T478K, E484A, F486S, Q498R, N501Y, Y505H, D614G , H655Y, N679K, P681H, N764K, D796Y, Q954H, N969K, D1199N |
| XBB | T19I, L24S, P25del, P26del, A27del, V83A, G142D, Y144del, H146Q, Q183E, V213E, G339H, R346T, L368I, S371F, S373P, S375F, T376A, D405N, R408S, K417N, N440K, V445P, G446S, N460K, S477N, T478K, E484A, F486S, F490S, Q498R, N501Y, Y505H, D614G, H655Y, N679K, P681H, N764K, D796Y, Q954H, N969K |
| XBB.1.5 | T19I , L24S, P25del, P26del, A27del, V83A , G142D, Y144del, H146Q, Q183E, V213E, G252V, G339H, R346T, L368I, S371F, S373P, S375F, T376A, D405N, R408S, K417N, N440K, V445P, G446S, N460K, S477N, T478K, E484A, F486P, F490S, Q498R, N501Y, Y505H, D614G, H655Y, N679K, P681H, N764K, D796Y, Q954H, N969K |
